# Magnetic Resonance Imaging and Magnetic Resonance Imaging Cholangiopancreatography of the Pancreas in Small Animals

**DOI:** 10.3390/vetsci9080378

**Published:** 2022-07-23

**Authors:** Chiara Briola

**Affiliations:** The Ralph Veterinary Referral Centre, Fourth Avenue Globe Business Park, Marlow SL71YG, UK; chiara.briola@gmail.com; Tel.: +39-3498459109

**Keywords:** MRI, MRCP, pancreas, endocrine, secretin, magnetic resonance imaging technique

## Abstract

**Simple Summary:**

In human medicine Magnetic resonance imaging (MRI) and MR cholangiopancreatography (MRCP) play a consistent role in the investigation of pancreatic and pancreatic duct disorders. In veterinary medicine the number of studies focused on MR and MRCP for pancreatic disease is scant, and the protocols are not yet standardized. This review will focus on the MRI and MRCP technical aspects of the protocols used for the investigation of pancreatic disease in veterinary medicine. The aim of this review is to elucidate the value and the potential of each MR and MRCP sequence listed in the different protocols, either in canine or feline patients, with the intention to build a valid and solid tool for further innovative studies.

**Abstract:**

Magnetic resonance imaging (MRI) and MR cholangiopancreatography (MRCP) have emerged as non-invasive diagnostic techniques for the diagnosis of pancreatic and pancreatic duct disorders in humans. The number of studies focused on MR and MRCP for pancreatic disease in small animals is very limited. MR has been described for the evaluation of insulinoma in dogs and to investigate pancreatitis in cats. The studies were based on a standard protocol with T2 weighted (w) fast recovery fast spin-echo (FRFSE) with and without fat suppression, T1w FSE pre-contrast and T1w FSE post-contrast with and without fat suppression. MRCP after secretin stimulation has been described in cats to assess the pancreatic ductal system, taking advantage of pulse sequences heavily T2w as rapid acquisition with rapid enhancement (RARE), fast-recovery fast spin-echo (FRFSE) sequences and single-shot fast spin-echo (SSFSE) sequences. In addition to the standard protocol, fast spoiled gradient recalled echo pulse sequences (fSPGR) and volume interpolated 3D gradient-echo T1w pulse sequences pre and post-contrast have also been used in cats, reaching the goal of assessing the biliary tree and the pancreatic duct with the same sequence and in multiple planes. Despite the small amount of data, the results show potential, and the most recent technical innovations, in particular, focused on diffusion MRI and fast acquisition, further support the need for continued evaluation of MRI as an effective instrument for the investigation of pancreatic disease.

## 1. Introduction

Magnetic resonance imaging (MRI) is considered one of the most important imaging modalities in human medicine for abdominal disease, and its value is constantly growing in the abdominal diagnostic routine workup. The absence of radiation exposure, the unique soft-tissue contrast, the three-dimensional capabilities and the most recent physiology-based MRI techniques are just an example of the advantages of MRI in comparison to computer tomography or ultrasound. In human medicine, magnetic resonance cholangiopancreatography (MRCP) has gained acceptance because it produces a recognizable projectional image format of the hepatic and pancreatic ducts in their native configuration. Furthermore, the combination of the administration of secretin and the dynamic evaluation of the duct’s diameter during the acquisition of MRCP sequences helps the visualization of the pancreatic ductal system and gives information about the pancreatic function, evaluating the outflow of the main pancreatic duct in correspondence with the lumen of the duodenum, and it is able to quantify the pancreatic exocrine fluid production [1].

Despite the advantages of the MRI previously mentioned, the necessity to perform the studies under general anesthesia and the requirement to use a high-field MR system with dedicated software has limited the role of MRI in veterinary medicine for the investigation of abdominal disease and, in particular, for pancreatic disease.

In 2019, Walczak et al. described the MRI findings in a group of dogs with pancreatic insulinomas [2], and in 2011 and 2012, Marolf et al. showed the potentiality of MR and MR cholangiopancreatography (MRCP) with and without secretin stimulation in normal feline patients and feline patients affected by pancreatitis [3,4]. Again, Marolf et al., in 2016, wrote about the state-of-the-art in computed tomography (CT) and MRI of the hepatobiliary system and pancreas [5].

The MR protocols used in the canine and feline patients were different, and the MRCP protocol with and without secretin stimulation was applied exclusively in the feline patient. This review will focus on the MRI technical aspects of the protocols used for the investigation of pancreatic disease in each different article in veterinary medicine. The aim of this review is to elucidate the value and the potential of each MR and MRCP sequence listed in the different protocols, either in canine or feline patients, with the intention to build a valid and solid tool for further innovative studies.

## 2. Methods

A systematic veterinary literature review was conducted using PubMed and Google Scholar to identify studies focused on MRI and MRCP of the pancreas either in canine or feline patients. The search strategy was performed by applying a combination of multiple query words, and no filter of the year of publication was applied in the process of the papers’ enrolment. The references of each article recalled, published in the English language, were successively manually checked with the aim of identifying any other pertinent articles not yet listed. The retrieved studies were filtered, and they were enrolled in the review only if the technical details of the MRI or the MRCP protocols were reported in the “material and methods” paragraph of the article.

## 3. Results

Seven papers matched the inclusion criteria; however, only three of them were included in this review; two of them were excluded from the review for ethical reasons [6,7], one of them [8] did not include any details about MRI or MRCP protocol, and one was already a review.

### MRI and MRCP Technique Details

All the acronyms used in this review are listed in Table 1.

The first paper analyzed from the technical point of view is “Canine insulinomas appear hyperintense on MRI T2-weighted images and isointense on T1-weighted images” [2].

An abdominal magnetic resonance imaging (MRI) study, which targeted the pancreas, was performed with the patients under general anesthesia using a 1.5 T superconducting magnet (GE Signa High-speed system, General Electric Medical Systems, Milwaukee, WI, U.S.). Four canine patients were enrolled in the study, and all of them were positioned in dorsal recumbency. Three of four patients had a range of weight between 15 and 43 kg, and their abdominal MRI study was acquired in a four-channel torso array coil. Only one patient, who weighed 7 kg, was small enough to be positioned in an extremity coil.

Regarding the geometrical parameters, all the sequences were acquired with a range of slice thickness between 3.5 and 4.0 mm, and with a maximum of a 0.5 mm slice gap. No technical details regarding matrix, number of excitations (NEX) and field of view (FOV) were mentioned in the article.

In three of four patients, the following protocol was acquired (Table 2).

MRI pulse sequences acquired before the administration of contrast medium:

- T1w FSE sequence in dorsal and transverse planes (echo time (TE) of 10.1–22.3 ms and repetition time (TR) of 300–700 ms);
- T2w FRFSE sequence in transverse and dorsal planes (TE 95.5–97.3 ms, TR 2550–4600 ms);
- T2w FRFSE sequence with fat suppression in transverse and dorsal planes (TE 91.3–97.3 ms, TR 2776–5200 ms);

Successively, a gadolinium-based contrast agent (gadopentetate dimeglumine, Magnevist, Bayer Healthcare Pharmaceuticals, Wayne, NJ, U.S.) was administered at a dose of 0.1 mmol/kg, and the following sequences were repeated:

- T1w sequence in the dorsal plane (TE and TR as previously reported);
- T1w sequence with fat suppression in the dorsal and transverse planes (TE 10.1–19.2 ms, TR 400–717 ms).

One of the patients had a computer tomography (CT) angiogram performed before the MRI study, and instead of the MRI postcontrast imaging, a STIR sequence in the transverse plane (TE 49.2 ms, TR 4750 ms, inversion time (TI) 150 ms) was acquired.

The second paper analyzed from the technical point of view is “Hepatic and pancreaticobiliary MRI and MRCP with and without secretin stimulation in normal cats” [3] in which Marolf et al. proposed a protocol with and without secretin administration for the investigation of the pancreas and pancreaticobiliary ducts in normal feline patients (Table 3).

As in the previously cited article, the abdominal MRI study was centered on the pancreatic region in the cranial abdomen, and all the patients underwent the MRI study under general anesthesia. The studies were performed using a 1.5 T superconducting magnet (GE Signa1.5 T, General Electric Medical Systems, U.S., Chicago, IL, USA).

All the feline patients were positioned in ventral recumbency to reduce to the minimum possible respiratory motion artifacts. All the studies were acquired with an extremity coil.

The details regarding the geometrical parameters (slice thickness and gap), matrix, number of excitations (NEX) and field of view (FOV) of each sequence cited from the article are reported between brackets in the bullet point below.

The following sequences were acquired before secretin administration and before contrast:

- T1w fSPGR in and out-of-phase in the transverse plane (TE 3.4 and 6 ms, TR 160, field of view (FOV) of 20 cm, slice thickness of 2.0 mm with a 0.2 mm gap, matrix of 256 × 192, and 1 number of excitations (NEX);
- T2w FSE with fat saturation sequence in dorsal and transverse planes. This specific sequence was acquired in the oblique sagittal plane with an orientation superimposable to the long axis of the pancreas in only one patient; in this specific case, flow compensation was applied, no phase wrap, TR 3050, TE 68 ms, FOV 18 cm, slice thickness 3.0 mm, 0.3 mm gap, matrix of 256 × 192, and 1 NEX;
- SSFSE with and without fat saturation in the transverse plane using flow compensation (TE 140 ms, TR at minimum), FOV of 20 cm, slice thickness 3.0 mm (0 mm gap), matrix of 256 × 256, and 0.53 NEX.

Before and after secretin administration, an MR cholangiopancreatography FRFSE sequence with flow compensation was performed in an oblique dorsal plane angled (TE 250 ms, TR 334, FOV 24 cm, slice thickness 3.0 mm with 0 mm of gap, matrix of 256 × 192, and 3 NEX). Intravenous secretin was then administered at 2 U/kg (0.2 mg/kg) to four of the five cats, and post-secretin images were obtained at approximately 3 min post-administration with the aim of assessing the pancreatic duct. MR cholangiopancreatography images were post-processed to create 3D maximum intensity projection (MIP) images.

A fast acquisition with multiphase Efgre (FAME) 3D SPGR post-secretin and pre- and post-gadolinium-based contrast agent injection (0.1 mL/kg) was acquired in the transverse and dorsal plane and also using an oblique sagittal oriented along the long axis of the pancreas (TE at minimum, FOV 22 cm, flip angle 15, slice thickness 1 mm, 60 images per slab matrix of 256 × 160, and 1 NEX).

The last paper analyzed from the technical point of view is “Magnetic resonance (MR) imaging and MR cholangiopancreatography findings in cats with cholangitis and pancreatitis”. The abdominal MRI study was centered on the cranial abdomen. All the patients underwent the MRI study under general anesthesia using a 1.5 T superconducting magnet (GE Signa1.5 T, General Electric Medical Systems, U.S.). As in the previously cited article of the same author, the patients were positioned in ventral recumbency in an extremity coil.

Here, Marolf et al. applied the previously listed MRI and MRCP protocols, excluding the single shot fast spin echo (SSFSE), which resulted in the previous paper not being diagnostic due to artifacts related to field inhomogeneities and an extremely low signal-to-noise ratio. For completeness, the sequences are listed below.

- T1 fast spin (FS) gradient echo (GRE) pre-and post-contrast in dorsal and transverse planes (instead of FAME 3D SPGR);
- T2 fat-saturated in dorsal and transverse planes;
- MRCP (T2w FR FSE) in dorsal planes;
- T1w fSPGR sequence in and out-of-phase in transverse planes.

Secretin was administered (as described in the article written by the same author and cited above) after the first MRCP sequence, and then, as in the previously mentioned protocol, the same MRCP sequence was repeated.

## 4. Discussion

Analyzing the sequences used in the aforementioned studies, it is evident that in veterinary medicine, there is not yet a solid MRI protocol for the investigation of pancreatic diseases. Furthermore, the MRCP protocols suggested in feline patients have not even yet been applied in canine patients.

Either in the canine or the feline MRI protocols, in the spectrum of the pulse sequences are listed the recent FR FSE T2w. With the aim of decreasing the acquisition imaging time without the loss of the T2w contrast, the major MR scanner manufacturers have expanded modifications of the standard FSE or TSE T2w sequences. The FR FSE T2w sequence is the modified FSE T2w technique created by GE Medical Systems. In this fast recovery modification, after the last echo of the FSE T2w echo train has been obtained, a further 180° pulse refocuses the residual magnetization in the transverse plane. After this, a −90° pulse is responsible for the protons flipping back to the longitudinal axis, preventing them from experiencing T1w recovery, causing an acceleration of the relaxation of the longitudinal magnetization, keeping the TR shorter without losing T2w contrast and leading to a reduction in the acquisition time. Based on a similar technique, Siemens and Philips created, respectively, RESTORE TSE and driven equilibrium radio frequency reset pulse (DRIVE) sequences [9]. In human beings, the usefulness of the application of FR FSE has been described in pediatric patients, in which very often it is not possible to apply the breath-holding technique commonly used in adult patients [9]. In adults, FR FSE T2w with the breath-holding technique showed a significant improvement in the signal-to-noise ratios and generated a higher contrast-to-noise ratio in the evaluation of hepatic lesions in contrast to the standard FSE T2w sequences [10,11].

In the initial protocol suggested by Marolf et al. for the investigation of the normal pancreas in feline patients, the single-shot fast spin-echo (SSFSE) was tested with and without fat saturation; however, this sequence resulted in not being diagnostic due to artifacts related to field inhomogeneities and low signal-to-noise ratio. FSE or TSE sequences are listed as RARE (rapid acquisition with relaxation enhancement) sequence types, originally described by Hennig et al. in 1986 [12]. The FSE or TSE can be acquired either in multishot or single-shot modes. When acquired in multishot modes, they can provide both T1 and T2w images with high spatial resolution; furthermore, it is also possible to add fat saturation pre-pulses. In comparison to the SE sequence, the fat shows a higher signal intensity, and the hepatic parenchyma appears more hypointense due to multiple refocusing of the radiofrequency (RF) pulses, which enhances the off-resonance magnetization transfer (MT) effect [13]. A single-shot version of FSE (SSFSE) can be acquired with the aim of producing extremely heavy T2w images in a very short time. However, the quality of a single-shot sequence is directly related to the application of a breath-holding technique, which, for obvious reasons, is not available in small animal patients. This restriction can potentially be overcome using MRI-compatible mechanical ventilation, which will allow sufficient time to acquire a single-shot sequence avoiding the presence of dedicated staff inside the Faraday cage; however, this solution is extremely expensive and not always applicable in veterinary medicine. A different solution is to apply techniques of motion suppression during free breathing as respiratory triggering, respiratory monitoring through navigator pulse techniques, and rotatory k-space sampling [14], which may help in reducing the artifacts.

Magnetic resonance cholangiopancreatography (MRCP) in human medicine was described for the first time in 1991 [15], and over the last three decades, it has matured noticeably, favoured by improvements in spatial resolution and time of acquisition. Nowadays, it has a consistent role as a non-invasive and capable method in the study of many biliary disorders, and it appears as a solid alternative to endoscopic retrograde cholangiopancreatography (ERCP) which is considered the gold standard in human medicine [16]. It makes use of heavily T2-weighted images, applying the previously mentioned T2w FRFSE sequences or their single-shot version (SSFSE), highlighting the intrinsic differences in the T2-weighted contrast between abdominal structures filled by immobile fluid (which are characterized by a long T2 relaxation time) and the other abdominal non-fluid-filled soft tissues (which are characterized by a shorter T2 relaxation time). This results in high signal intensity of static or slow-moving fluids within the biliary tree and pancreatic duct associated with a lower signal intensity of the surrounding hepatic and pancreatic soft tissues, in particular, when post-processed as maximum intensity projections (MIP). In a normal feline patient, the maximum width of the pancreatic duct visualized ultrasonographically was 0.13 ± 0.04 cm (0.06–0.24 cm) [17]. In a normal canine patient, the mean pancreatic duct diameter visualized ultrasonographically was 0.6 ± 0.2 mm in the left lobe and 0.7 ± 0.2 mm in the right lobe [18]. In MRCP the visualization of normal calibre pancreatic ducts is directly correlated to the volume of fluid and obviously also to the inherent dimension of the pancreatic duct; for this reason, the MRCP protocol takes advantage of the administration of secretin to maximize the distension and therefore the detection of the pancreatic ducts.

Secretin is a polypeptide hormone produced by duodenal mucosa in response to the presence of acid in the intestinal lumen. The role of the secretin is to stimulate the pancreas to secrete water and bicarbonate, resulting in an increment of the volume of fluid within the duct, which becomes more easily identified [3,19,20,21]. Due to its inherent ability to cause an increase in the volume of fluid in the pancreatic ducts, secretin can be used as a target contrast medium in MRCP studies and contributes to reducing the gap between the diagnostic capabilities of MRCP and the more invasive, even if still currently the gold standard, ERCP [1].

Parallel to the SE and FSE sequences listed in the different protocols, the gradient echo (GRE) pulse sequences are also present, which are based on the utilization of gradients instead of multiple RF pulses to produce the echo. Taking advantage of the absence of multiple RF pulses, the GRE can be acquired with a significant reduction in the scan time (becoming a feasible breath-holding technique sequence in human medicine). There are two different groups of GRE sequence based on the presence or not of the coherence of transverse magnetization.

The first group of GRE sequences is made by the coherent steady-state (not spoiled), the so-called fast imaging with steady-state precession (FISP); however, due to their high sensitivity to motion artifacts, they are not widely used in pancreatic MR imaging.

The second group is made by the GRE noncoherent steady-state (which included either the T1w spoiled gradient echo 2D or the 3D version FAME SPGR listed in the previously mentioned protocols). The term “spoiled” gradient echo is synonymous with “incoherent gradient echo” and spoiled means to ruin or destroy something, which is what this sequence is doing to the residual transverse magnetization [22]. This type of sequence can also be acquired in and out of phase depending on the TE used. A GRE sequence acquired in and out of phase is a paired sequence acquired with the same TR but with two different TE. This sequence is based on chemical shift imaging, which is founded on the intrinsic different resonant frequency of the protons in water/fluid and fat tissue. The sequences that are taking advantage of the chemical shift can be targeted to acquire the amount of signal either from water or fat protons when their signal is either in phase or out of phase, giving in this way the opportunity to determine the quantity of water signal and fat signal in every single voxel. Therefore, the specific loss of signal intensity obtained comparing the in-phase sequence to the out-phase sequence gives information about the amount of fat inside the voxel, becoming a tool, for example, to define the tissue composition of parenchymal organs and/or masses. For this reason, the GRE noncoherent steady-state sequences can play a significant role in the diagnosis of a wide spectrum of benign or aggressive diseases either in the thoracic or abdominal cavity [23,24]. Manufacturer-specific examples of GRE noncoherent 2D sequences are T1 FFE (Philips), SPGR (GE) and FLASH (Siemens). 

Parallel to the 2D GRE noncoherent sequences, the 3D version is also available, which is able to perform further interpolation through the slice selection direction. In human medicine, this sequence is nowadays commonly used for hepatic, renal and pancreatic dynamic contrast studies, and the old 2D FLASH has been replaced by the new so-called volume interpolated body examination (VIBE) sequence (Siemens) or fast acquisition with multiphase elliptical fast gradient echo (FAME Efgre) sequence (GE). The most important reason for this change is the opportunity to acquire a nearly isotropic 3D sequence; however, these sequences are highly sensitive to motion artifacts and may result in poor performance, especially without a breath-holding technique. Another reason for acquiring a 3D sequence instead of 2D is the novel application of this kind of sequence in contrast-enhanced MR angiography (MRA); it can be performed either with or without fat saturation pre-pulse, and it takes advantage of its capability to provide T1w images with thin slices [1,25,26,27,28,29]. Last but of course not least, this sequence is T1w and allows acquiring a nearly isotropic 3D T1w GRE sequence pre- and post-contrast administration.

**Figure 1:**
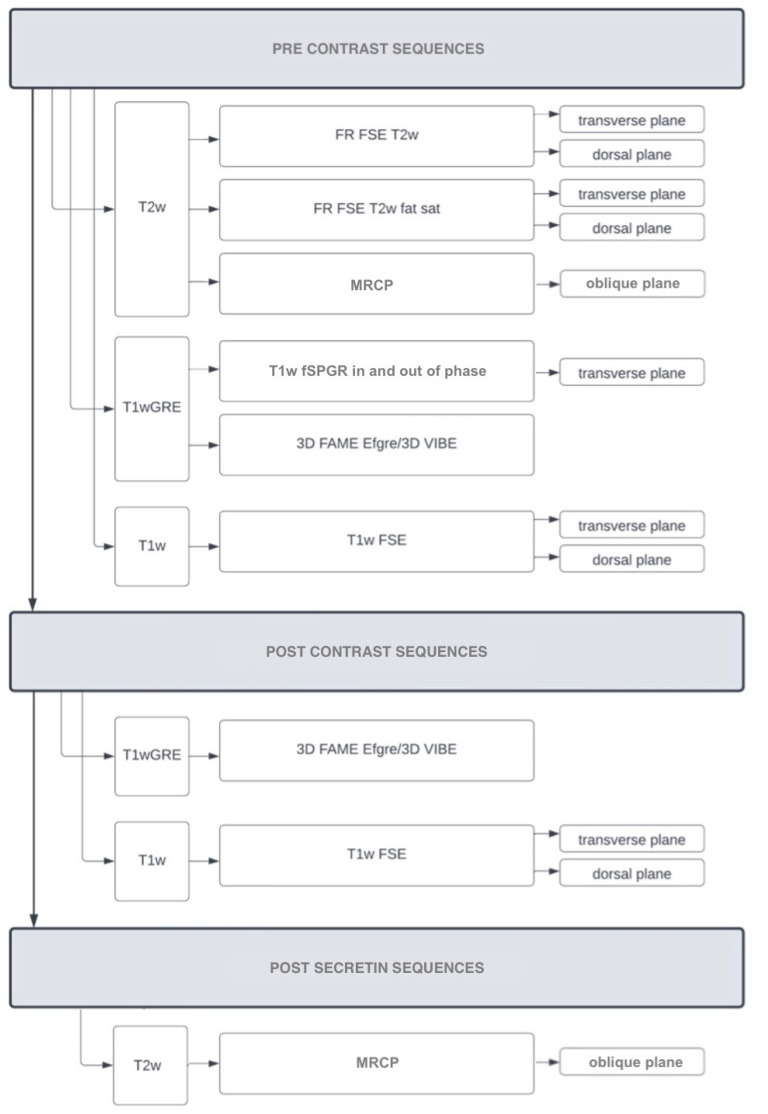
MRI and MRCP proposed protocol for canine and feline patients, including pre-contrast sequence, post-contrast sequences and post-secretin sequence.

To help the reader to differentiate between the spectrum of the sequences discussed, in Figure 1, a protocol is suggested that could potentially be applied in future studies. The order of the sequences or the kinds of sequences in the suggested protocol is not written in stone, and further studies are needed to standardize a solid protocol.

## 5. Conclusions

In the author’s opinion, the complexity of this field, the wide spectrum of sequences available, the unavoidable necessity to perform the MR study under general anesthesia, and the lack of standard protocols evaluated either in canine or feline patients may be responsible for the profound gap between human and small animal medicine in pancreatic MR, but in general, abdominal MR. However, especially based on the growing number of high-field MR scanners in small animal veterinary medicine, and also in light of the most recent innovative techniques, MR and MRCP have the potential to become consistent, valid and non-invasive diagnostic imaging tools.

Two innovative sequences could represent further directions in pancreatic MR in small animals: the first one is the dynamic, multiphase contrast-enhanced MR, and the second is the diffusion-weighted imaging (DWI).

In the MR pancreatic protocol in human medicine, there is a growing interest in dynamic, multiphase contrast-enhanced MR. Controlled aliasing in parallel imaging results in higher acceleration factor (CAIPIRINHA)-time-resolved angiography with interleaved stochastic trajectories (TWIST)-VIBE (CDT-VIBE) is an innovative contrast-enhanced T1w 3D fat suppression GRE sequence. In human medicine, this sequence has shown potential because it is founded on the ability to acquire multiple subphases during the same arterial phase in the time of a single breath-hold. It allows to dynamically characterize the vascular behaviour of a focal lesion, and for this reason, it can play a role in the investigation of focal hypervascular hepatic lesions. Regarding pancreatic diseases, TWIST-VIBE can help in the identification of small hypervascular (in the arterial phase) lesions, for example, small neuroendocrine tumours such as insulinoma [25,26,27,28,29]. Further studies in small animals are necessary to estimate the potential and the clinical application of this new technique in the evaluation of hepatic and pancreatic disease (e.g., pancreatic insulinoma) [26,27,28,29].

In diffusion-weighted imaging, contrast is the result of the different movement of water molecules (random Brownian motion) that is intrinsically related to their bonds in a different kind of tissue (passage of water molecules from intra and extracellular spaces and vessels). In each organ, water molecules are not able to move freely and randomly because they are constrained by the organization of the tissues in which they live. If the inherent boundaries specific to each tissue are disrupted or if the cellularity decreases, the opportunity of movement for the water molecules will increase, generating an increment in the so-called water diffusion coefficient.

For these reasons, various pathological conditions, such as neoplasia, acute or chronic inflammation, granuloma/abscess, fat infiltration, fibrosis and ischemic or hemorrhagic infarct, can influence and modify the water diffusion and, secondarily, the appearance of the organ in DWI sequences and generate different DWI derived apparent diffusion coefficient (ADC) values.

In human medicine, DWI has started to play a significant role in oncology, especially in the differentiation between malignant ductal adenocarcinoma and focal pancreatitis. In veterinary medicine, DWI can potentially be of particular interest in the investigation of chronic pancreatitis either in the canine or feline patient or in the differentiation between benign pancreatic cystic conditions or necrosis associated or not with pancreatic neoplasia [30,31,32,33,34,35,36].

## Figures and Tables

**Table 1:** All the acronyms used in the review are listed in the first column, with their respective extended description in the second column.

Acronym	Description
ADC	Apparent Diffusion Coefficient
CAIPIRINHA	Controlled Aliasing In Parallel Imaging Results In Higher Acceleration Factor
DWI	Diffusion Weighted Imaging
Efgr	Elliptical fast gradient echo
ERCP	Endoscopic Retrograde Cholangiopancreatography
FAME	Fast Acquisition with Multiphase Efgr
FISP	Fast Imaging with Steady State procession
FLASH	Fast Low Angle Shot
FOV	Field of View
FRFSE	Fast Recovery Fast Spin Echo
FSE	Fast Spin Echo
fSPGR	Fast Spoiled Gradient Recalled Echo Pulse Sequences
GRE	Gradient Echo
MPI	Maximum Intensity Projections
MRA	MR Angiography
MRCP	MR cholangiopancreatography
MRI	Magnetic Resonance Imaging
NEX	Number of Excitations
RARE	Rapid Acquisition with Rapid Enhancement
RF	Radiofrequency pulse
SSFSE	Single Shot Fast Spin Echo
TWIST	Time-resolved angiography With Interleaved Stochastic Trajectories
VIBE	Volume Interpolated Body Examination

**Table 2:** MRI and MRCP protocols, including technical details used in Walczak et al., 2019. “Dor” stays for dorsal, “tra” for transverse, “sag” for sagittal, and “obl” for oblique.

Walczak et al., 2019, 1.5T GE									
Pre-Contrast	Planes					TE ms	TR ms	Thickness mm	Gap mm
	Dor	Tra	Sag	Obl	3D				
T1w FSE	✓	✓				10.1–22.3	300–700	3.5–4.00	0.5
T2w FRFSE	✓	✓				95.5–97.3	2500–4600	3.5–4.00	0.5
T2w FRFSE fat sat	✓	✓				91.3–97.3	2776–5200	3.5–4.00	0.5
**Post-Contrast**									
T1wFSE	✓	✓				10.1–22.3	300–700	3.5–4.00	0.5
T1w FRFSE fat sat	✓	✓				10.1–19.2	400–717	3.5–4.00	0.5

**Table 3:** MRI and MRCP protocols, including technical details used in Marolf et al., 2011. “Dor” stays for dorsal, “tra” for transverse, “sag” for sagittal, “obl” for oblique, “Min” for minimum, and “Thick” for thickness.

Marolf et al., 2011, 1.5T GE												
Pre-Contrast	Planes					TE ms	TR ms	Thick mm	Gap mm	Matrix	NEX	FOV (cm)
	Dor	Tra	Sag	Obl	3D							
T1w fSPGR (in and out of phase)		✓				3.4	160	2.0	0.2	256 × 192	1	20
T2w FSE fat sat	✓	✓		✓		68	3050	3.0	0.3	256 × 192	1	18
SSFSE		✓				140	Min	3.0	0	256 × 256	0.53	20
SSFSE fat sat		✓				140	Min	3.0	0	256 × 256	0.53	20
MRCP FRFSE				✓		250	334	3.0	0	256 × 192	3	24
FAME 3D SPGR					✓	Min		1.0		256 × 160	1	22
**Post-Contrast**												
FAME 3D SPGR					✓	Min		1.0		256 × 160	1	22
**Post-Secretin**												
FAME 3D SPGR					✓	Min		1.0		256 × 160	1	22
MRCP FRFSE				✓		250	334	3.0	0	256 × 192	3	24

## Data Availability

Not applicable.
